# Retrovirus Integration Database (RID): a public database for retroviral insertion sites into host genomes

**DOI:** 10.1186/s12977-016-0277-6

**Published:** 2016-07-04

**Authors:** Wei Shao, Jigui Shan, Mary F. Kearney, Xiaolin Wu, Frank Maldarelli, John W. Mellors, Brian Luke, John M. Coffin, Stephen H. Hughes

**Affiliations:** Advanced Biomedical Computing Center, Leidos Biomedical Research, Inc., Frederick National Laboratory for Cancer Research (FNLCR), Frederick, MD USA; HIV Dynamics and Replication Program, NCI, Frederick, MD USA; Leidos Biomedical Research, Inc., Frederick National Laboratory for Cancer Research (FNLCR), Frederick, MD USA; Division of Infectious Disease, University of Pittsburgh, Pittsburgh, PA USA; Department of Molecular Biology and Microbiology, Tufts University, Boston, MA USA

**Keywords:** Retrovirus, HIV, Integration site, Database, Integration site assay, ISA, Expanded clones

## Abstract

The NCI Retrovirus Integration Database is a MySql-based relational database created for storing and retrieving comprehensive information about retroviral integration sites, primarily, but not exclusively, HIV-1. The database is accessible to the public for submission or extraction of data originating from experiments aimed at collecting information related to retroviral integration sites including: the site of integration into the host genome, the virus family and subtype, the origin of the sample, gene exons/introns associated with integration, and proviral orientation. Information about the references from which the data were collected is also stored in the database. Tools are built into the website that can be used to map the integration sites to UCSC genome browser, to plot the integration site patterns on a chromosome, and to display provirus LTRs in their inserted genome sequence. The website is robust, user friendly, and allows users to query the database and analyze the data dynamically. Availability: https://rid.ncifcrf.gov; or http://home.ncifcrf.gov/hivdrp/resources.htm.

## Background

For a retrovirus to replicate, the virus must integrate a DNA copy of its genome, producing a provirus in the genome of the infected host cell. Research into host integration sites of retroviral genomes has been on-going for many years [2, 8, 13, 14]. Insertion into regions near host genes can affect the expression of the host gene. If the host gene has an important role in controlling cell growth and division, integration can cause clonal cell expansion, and may be involved in the development of malignancy [1, 12, 15, 18].

**Fig. 1:**
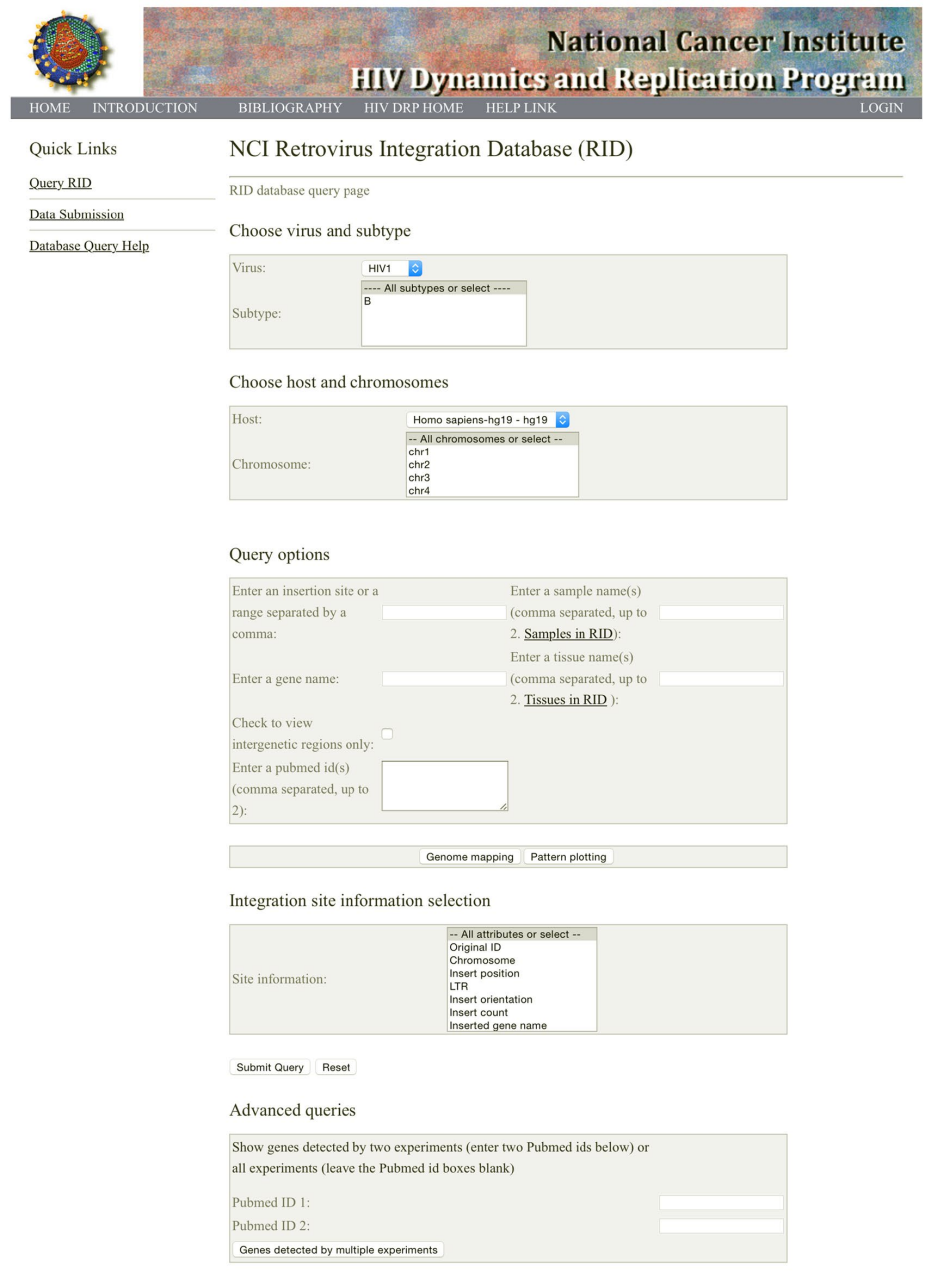
Screen shot of the RID home page

The advent of next generation sequencing technologies has allowed for tens of thousands or even millions of retroviral integration sites to be obtained in single experiments [5, 10, 16, 19, 20]. Currently, integration site information must be downloaded from the supplementary files of publications or obtained from the investigators directly, making collection time consuming and difficult. Recently, there has been a rapid increase in the amount of retroviral integration site data that is available, and there is a need for a readily accessible database to store, retrieve, and analyze integration site data. In addition, a public integration site database will allow concurrent mapping and reporting of proviral orientation across and among studies, and can help to avoid issues that can arise when integration sites are mapped using different genome builds or by applying different definitions for the orientation of the gene or the provirus. For example, Maldarelli et al. and Wagner et al. mapped integration sites to the human genome build hg19 [12, 18], whereas Ikeda et al. and Wang et al. mapped their integration sites to an older genome build [9, 19]. Furthermore, LaFave et al. and Wagner et al. defined “+” proviral orientation as being in the same orientation as the chromosome [10, 18], whereas Han et al. [7] and Sunshine et al. [17] defined “+” proviral orientation as being the same as the target gene. To avoid such inconsistencies and to facilitate the storage, retrieval, and coordinated analyses of published retroviral integration site data, we built the NCI Retrovirus Integration Database (RID) (https://rid.ncifcrf.gov/, Fig. 1) and are making it available for public use.

## Methods

We collected retrovirus integration sites information from published papers or by directly contacting the authors when the information that was not readily available in the published papers (see acknowledgements). For consistency, we only extracted host, chromosome, integration site, virus type or subtype, proviral orientation, and LTR from those datasets and then we performed gene mapping (including intron/exon mapping) using NCBI genome. This local gene annotation database is derived from NCBI genomes (http://www.ncbi.nlm.nih.gov/genome/). If an integration site is not in a gene, then the nearest genes in both directions were mapped and stored in RID. All gene annotations were based on human genome build GRCH37/hg19. For the raw data using older genome builds, the integration sites were converted to hg19 using LiftOver, a genome converting tool provided by UCSC Genome Bioinformatics (http://genome.ucsc.edu/cgi-bin/hgLiftOver). Proviruses orientations have been converted to a uniform standard: if a provirus is integrated in the same orientation as the target chromosome (using the UCSC numbering convention), it is defined as “+”, otherwise, it is defined as “−”.

## Results

RID provides a common place to store and retrieve information describing retroviral integration sites. It is intended for public use and requires no login information. The database stores information on the sites of retroviral integrations into host genomes, the host type, virus type and subtype, a description of the sample origin, such as tissue type, and the reference from which the data originated. The integration site information is presented in a table that includes the host chromosome number, the specific coordinates of integration, the nearest gene, whether the integration site was identified from the retroviral 5′LTR or 3′LTR; and, if the integration site is in a gene, whether it is in an exon or an intron. Currently, RID includes valid data from retroviral insertion sites of HIV-1, HTLV-1, and MLV from multiple publications [4, 5, 7, 9–12, 14, 16, 18] and the database is intended to include integration site information from other retrovirus as more data become available. All of the data in RID have been mapped to a recent completely annotated genome build for the specific host, for example, human genome hg19 for HIV-1 and HTLV-1.

### Accessing information on the database

The database can be accessed using current version of web browsers including Internet Explorer, Chrome, Firefox, and Safari. It is compatible with PC, Mac, iPad, and cellphones. The main menu for the RID web interface is divided into five sections (Fig. 1): Choose virus and subtype, Choose host and chromosomes, Query options, Integration site information selection, and Advanced queries. The main menu allows users to access data by searching for integration sites for a specific virus or a specific viral subtype in the “Choose virus and subtype” section. Users then can access the data by selecting a specific host type and one or all of the chromosomes from “Choose host and chromosomes” section. Users can then select the “Submit Query” button to display the query result.

Users can limit their query by choosing an option in the “Query option” section. For example, a nucleotide position range on a specific chromosome can be chosen to search for integration sites within a specific region of the host genome or users can search query integration sites based on genes, the PubMed ID of one or two specific publications, or a sample name or a tissue type to narrow the query. The “ADVANCED QUERIES” section can be used to find integrations that have been reported in the same genes across multiple studies.

**Fig. 2:**
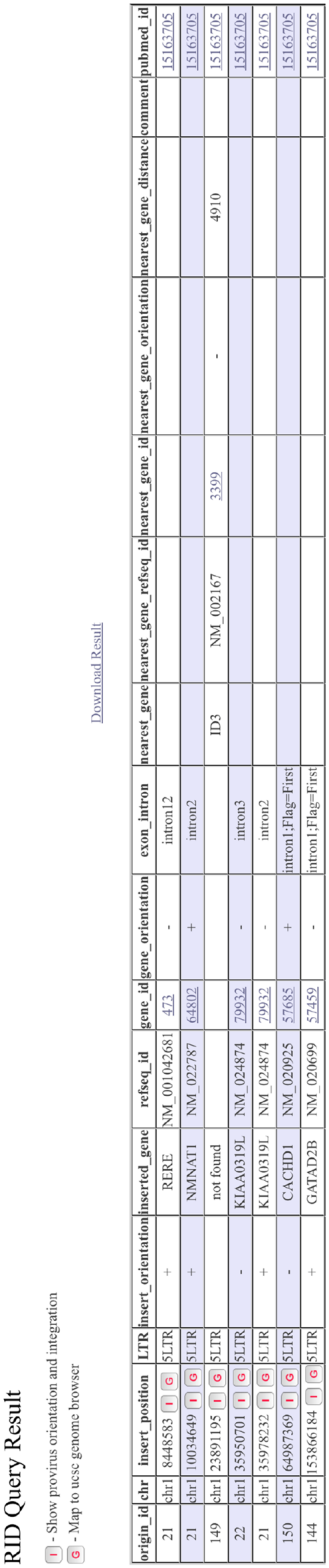
Partial screen shot of query results from the RID. In each *row*, clicking buttons “I”, “G”, or hyperlinks for gene_id, and pubmed_id can be used to link the integration site being investigated to the corresponding host genome sequence, host genome mapping, gene information, and PubMed abstract

**Fig. 3:**
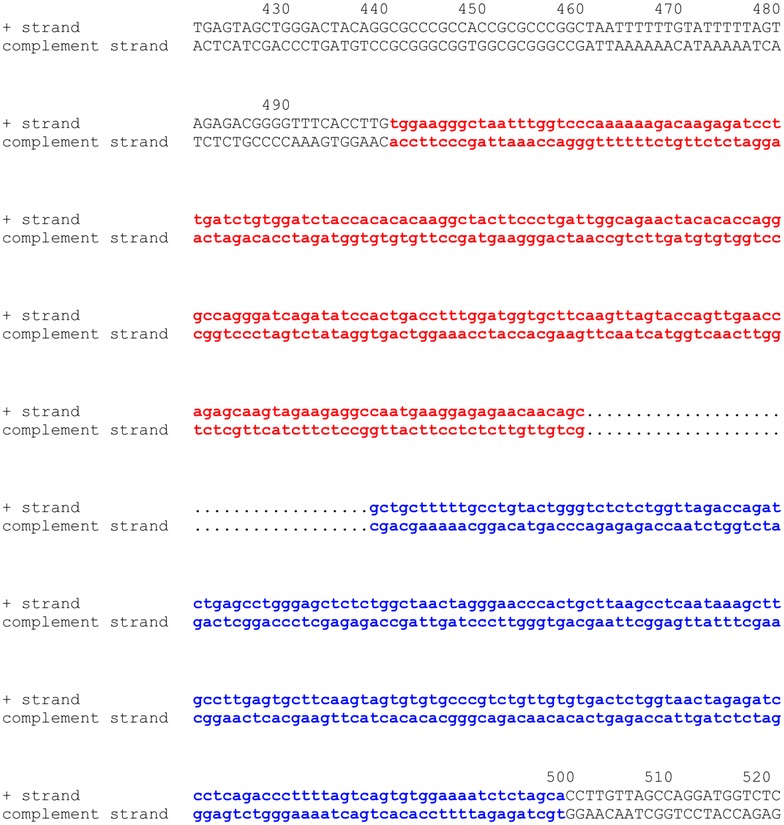
Partial screen shot of human chromosome 16 position 14307633 where a provirus is inserted [12] and the flanking host genomic sequences. *Red color* shows a portion of the 5′ LTR of HIV-1 pNL4-3. *Blue color* shows a portion of the 3′ LTR of HIV-1 pNL4-3

**Fig. 4:**
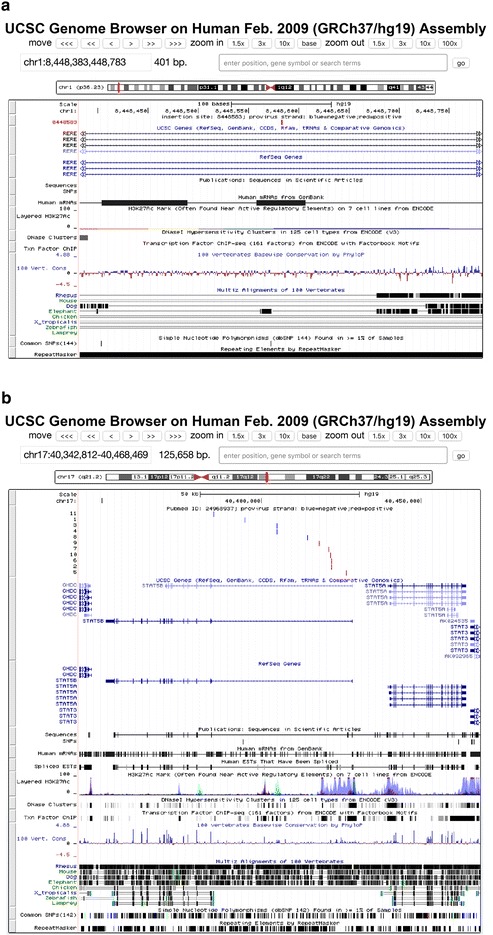
Integration sites mapped using the UCSC genome browser. *Red vertical bars* show HIV-1 proviruses in the positive orientation relative to the conventional chromosome numbering while *blue vertical bars* show proviruses in the negative orientation. **a** Screen shot from the UCSC genome browser showing the position of an integration site in the RERE gene on human chromosome 1. **b** Screen shot from the UCSC genome browser showing all integration sites in STAT5B gene reported by Maldarelli et al. [12]

The results of any search can be exported, as a text file (Fig. 2), for inclusion in presentations or publications. After obtaining query results, users can click the “I” button on the results page (Fig. 2) to display the chromosome information for the integration sites including the sequence data for the 500 host nucleotides flanking the integration site (Fig. 3) joined to a fragment of nucleotides at each end of the consensus LTR for the virus chosen. It also shows the correct length of the target site duplication depending on the virus; for example, for HIV-1, it shows five nucleotide duplications, for HTLV-1, it shows six nucleotide duplications, and for MLV, it shows four nucleotide duplications at each end the provirus [2]. In this display, the 5′LTR is highlighted in red and the 3′LTR in blue. Users can also click the “G” button on the results page (Fig. 2) to display a particular integration site relative to the full chromosome on the UCSC genome page (https://genome.ucsc.edu/, Fig. 4a) or they can click the hyperlink to “gene_id” to display the detailed gene information from the NCBI Gene database (http://www.ncbi.nlm.nih.gov/gene/). The “pubmed_id” link will provide the corresponding paper from NCBI PubMed.

**Fig. 5:**
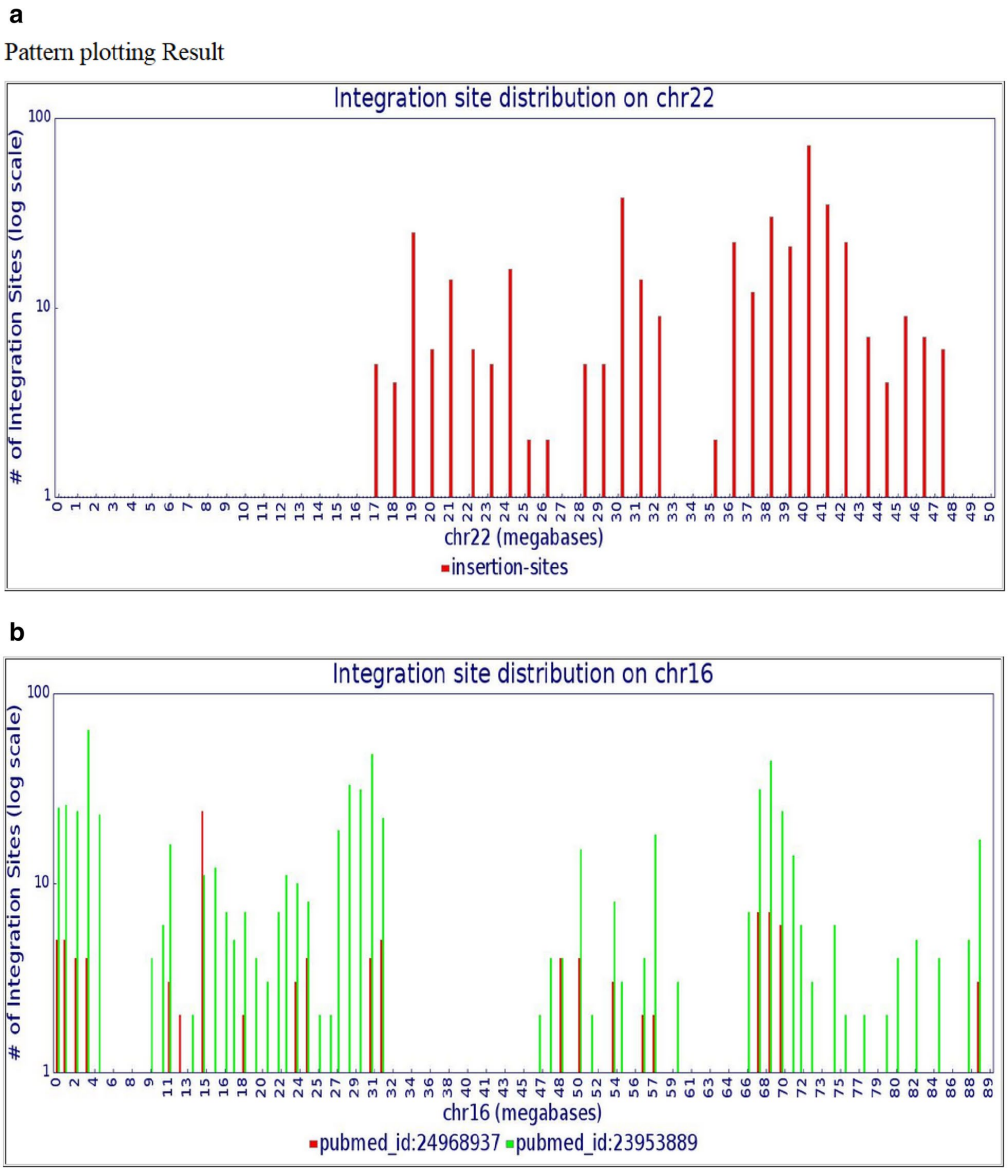
Distribution of integration sites, presented in bins of 1 million nucleotides, along a chromosome. The *Y axis* shows the number of HIV-1 integration sites in 1-megabase bins. The *X axis* shows the positions in megabases. **a** Distribution of all HIV-1 subtype B integration sites stored in RID for human chromosome 22. **b** Distribution comparison between two publications (*red color*: [12] and *green color*: [14]) indicated by PubMed IDs. The *vertical arrow* indicates the position of the MKL2 gene, a region of selected integration sites reported by Maldarelli et al. [12]

RID also includes tools to show the distribution of integration sites along a chromosome. After choosing a chromosome, users can click the “Genome mapping” button to display all the integration sites mapped to a specific chromosome in UCSC genome browser. They can also combine three query options; for example, chromosome 17, gene name STAT5B, and Pubmed_id 24968937 [12], to map the integration sites in STAT5B in UCSC genome browser (Fig. 4b) or they can click “Pattern plotting” to display the distribution of the integration sites on specific chromosomes in 1 million nucleotide bins (Fig. 5a) by, for example, selecting chromosome 22. Note that no integration sites in chromosome 22 are seen in the first 14 million bases or so, reflecting the fact that chromosome 22 is one of the five human acrocentric chromosomes. The centromere is at 14.7 million bp in length. The short arm is rich in tandem repeats [6] and has not been accurately sequenced or annotated. Such sequencing gaps still exist near the centromeres of all chromosomes [3] which make the discovery of integration sites in these region difficult. The RID tools can also be used with the nucleotide, gene name, PubMed ID, sample, or tissue type selections to display integration site distributions based on these parameters. For example, Fig. 5b shows the comparison of integration site distribution patterns on chromosome 16 from two studies [12, 18].

### Uploading data to the database

Users are encouraged to submit their published data to RID. The detailed submission instruction and templates can be accessed in Data Submission tab (Fig. 1). Generally speaking, only data from published peer-reviewed studies will be accepted and made available on the website. We reserve the right not to post data if inspection of the submitted data shows that there are obvious problems with the dataset. In that case, we would contact the authors for clarification.

## Conclusion

We have built a large scale, robust relational database called the Retroviral Integration Database (RID) which will be used to store publically available retrovirus integration site data. Users can query all available integration sites or specifically analyze integration sites in specific chromosomes, genes, tissues, etc. Several useful tools are built into the website that are designed to help map integration sites to the UCSC genome browser, to plot integration sites on particular chromosomes, and to determine the flanking host sequences. This database can be used to facilitate meta-analyses of retrovirus integration sites and their chromosomal distribution.
